# Multi-Omic Characterization of Single Cells and Cell-Free Components Detected in the Cerebrospinal Fluid of Patients with Leptomeningeal Disease

**DOI:** 10.3390/cancers16223746

**Published:** 2024-11-06

**Authors:** Stephanie N. Shishido, Amelia Marvit, Doanna Pham, Theresa Luo, Liya Xu, Jeremy Mason, Saul J. Priceman, Jana Portnow, Peter Kuhn

**Affiliations:** 1 Convergent Science Institute for Cancer, Michelson Center, University of Southern California, Los Angeles, CA 90089, USAdmpham@usc.edu (D.P.); luothere@usc.edu (T.L.); masonj@usc.edu (J.M.); 2 The Vision Center at Children’s Hospital Los Angeles, Los Angeles, CA 90027, USA; liyaxu@usc.edu; 3 USC Roski Eye Institute, Keck School of Medicine, University of Southern California, Los Angeles, CA 90033, USA; 4 Institute of Urology, Catherine & Joseph Aresty Department of Urology, Keck School of Medicine, University of Southern California, Los Angeles, CA 90033, USA; 5 Department of Hematology and Hematopoietic Cell Transplantation, City of Hope, 1500 East Duarte Road, Duarte, CA 91010, USA; priceman@usc.edu; 6 Department of Medicine, KSOM/NCCC Center for Cancer Cellular Immunotherapy, Keck School of Medicine of USC, Los Angeles, CA 90033, USA; 7 Department of Medical Oncology & Therapeutics Research, City of Hope, 1500 East Duarte Road, Duarte, CA 91010, USA; jportnow@coh.org; 8 Norris Comprehensive Cancer Center, Keck School of Medicine, University of Southern California, Los Angeles, CA 90033, USA; 9 Department of Aerospace and Mechanical Engineering, Viterbi School of Engineering, University of Southern California, Los Angeles, CA 90089, USA; 10 Department of Biological Sciences, Dornsife College of Letters, Arts, and Sciences, University of Southern California, Los Angeles, CA 90089, USA; 11 Department of Biomedical Engineering, Viterbi School of Engineering, University of Southern California, Los Angeles, CA 90089, USA

**Keywords:** liquid biopsy, cerebrospinal fluid, leptomeningeal disease, circulating tumor cells, brain metastasis

## Abstract

Background/Objectives: Up to 30% of patients with breast cancers will develop brain or leptomeningeal metastases, and this risk is especially high with HER2-positive cancers. For patients with central nervous system metastases, cerebrospinal fluid (CSF) liquid biopsies are a promising opportunity to monitor disease, inform treatment, and predict prognosis. This pilot study investigated CSF liquid biopsy analytes from three patients diagnosed with central nervous system metastases based on imaging but not confirmed via clinical cytology. Methods: The detection of cellular analytes with the non-enrichment high-definition single-cell assay (HDSCA3.0) workflow was compared between the CSF and matched peripheral blood (PB) samples. Results: Circulating tumor cells (CTCs) were detected in the CSF but not the PB and were subsequently molecularly characterized using single-cell genomics and targeted multiplexed proteomics to reveal a clonal population of phenotypically heterogeneous cells. There was a lack of concordance in the copy number alteration profiles between CTCs and cell-free DNA (cfDNA) in the CSF. Extracellular vesicle surface marker analysis in CSF revealed a prominent signal among tetraspanins (CD9/CD63/CD81), with CD81 exhibiting the highest signal across all patients. Conclusions: The data presented suggest that CSF could be a useful tool for diagnosing and assessing disease severity.

## Simple Summary

Almost a third of breast cancer patients may develop cancer that spreads to the brain or the tissue around it, with a higher risk for those with HER2-positive cancers. For these patients, using cerebrospinal fluid (CSF) as a liquid biopsy is a promising way to track the disease, guide treatment, and predict outcomes. This study looked at CSF samples from three patients with brain metastases (seen on scans but not confirmed by lab tests) and compared them to blood samples. Cancer cells were found in the CSF, but not in the blood, and were further studied to reveal a group of cancer cells that looked different from one another. Overall, the results suggest that CSF could be a useful tool for diagnosing and tracking cancer in these patients.

## 1. Introduction

Brain and leptomeningeal metastases are a major challenge in the management of breast cancer (BC), affecting 10–16% of living advanced BC patients [1,2] and up to 30% in autopsy cases [3]. Approximately 2–12% of patients with metastatic lesions in the brain may have leptomeningeal disease (LMD) at the time of initial intracranial presentation, with up to 37% developing LMD later in the clinical course [4,5,6,7]. LMD is distinguished by the progression of disease into the leptomeninges and cerebrospinal fluid (CSF). Patients with LMD have a poor prognosis—the median survival being only 2–4 months—with treatment extending patient survival to 6 months or longer [8,9,10,11]. CSF cytology is a component for assessing disease progression, along with MRI and patient symptoms. MRI is the standard imaging modality for LMD, but has notable limitations with detecting small lesions and differentiating between a malignancy and other conditions like inflammatory or infectious processes, thereby complicating diagnosis [12]. CSF is a typically paucicellular liquid in healthy individuals but has been shown to contain malignant cells in patients with LMD and brain tumors. Its limitations, including the need for a large sample volume, subjectivity, low sensitivity and specificity (range from 50–60% and 75–80%, respectively), and potential for ambiguous results [13,14], underscore the need for new tools to detect and monitor brain metastases and primary brain tumors.

Applying methods of the liquid biopsy to CSF collections could be promising for informing and monitoring disease progression, as it could extend the sensitivity and specificity of current clinical practice. Studies have shown that liquid biopsy techniques can achieve higher sensitivity, identifying LMD in cases where MRI and cytology fail [15,16]. Single-cell molecular characterization can enhance our understanding of tumor heterogeneity, clonality, and treatment responses compared to tissue biopsies alone, which are limited to a single spatiotemporal point. Combined with genomic analysis of circulating cell free DNA (cfDNA), a comprehensive liquid biopsy can provide a more complete representation of intratumor heterogeneity, which can better inform clinical choices around targeted therapies. In the context of brain tumors, a peripheral blood liquid biopsy may not be the right approach to sample tumor analytes confined within the blood brain barrier. Additionally, the less invasive nature of CSF over tissue biopsies allows for longitudinal sampling, which enables the monitoring of cancer related events over time. If an indwelling intraventricular reservoir or catheter system is in place, then longitudinal sampling of CSF can be obtained to assess the status of central nervous system disease over time. This longitudinal approach becomes invaluable in challenging scenarios, such as brain metastases, providing a means to assess therapeutic responses and resistance throughout treatment.

The third generation high-definition single-cell assay (HDSCA3.0) workflow provides a non-enrichment approach to cellular and acellular molecular analysis. Previous studies with this workflow have shown that enumeration of circulating tumor cells (CTCs) and cfDNA are strong prognostic markers in multiple cancer types [17,18]. In the study presented here, we show the utility of the CSF as a liquid biopsy to detect and characterize CTCs in BC patients with brain or leptomeningeal metastases. We compare the single cells detected in the CSF to the rare cells detected in the peripheral blood (PB), with further molecular characterization of the CSF via multi-omic analysis of CTCs and genomic analysis of cfDNA. The data presented support the CSF liquid biopsy’s utility in disease detection and monitoring, offering a platform for further molecular analysis to elucidate the biology and significance of tumor cells.

## 2. Materials and Methods

### 2.1. Study Design

**Figure 1:**
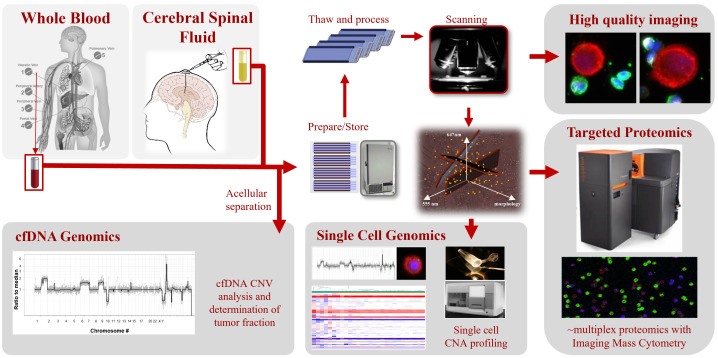
The HDSCA3.0 workflow for PB and CSF. Patient samples are processed, separating the acellular from the cellular fractions prior to cryopreservation. Cells that have been plated on custom glass slides are stained with immunofluorescent antibodies, slides are scanned, and images computationally analyzed for candidate events. Single cells and oncosomes are further imaged at high-resolution and may be molecularly characterized by single-cell genomics or targeted multiplexed proteomics. The acellular fraction is analyzed for cfDNA and EVs.

Three patients with metastatic BC from NCT03696030 had PB and CSF samples collected at a single timepoint prior to therapy for liquid biopsy analysis using the HDSCA3.0 workflow. All patients had primary BC and now present with a HER2-positive malignancy within the central nervous system. CSF was collected through a Rickham reservoir that was connected to a catheter placed in the patient’s lateral cerebral ventricle. Clinical CSF cytology was conducted on a corresponding sample collected either on the same day (Patients 1 and 3) or within 2 days of collection (Patient 2). Details of the workflow with downstream analyses are shown in Figure 1. With a detection limit of approximately 1 cell per 3 million, the HDSCA workflow has been extensively validated for PB in several large studies [19,20,21,22,23,24,25,26,27,28] and has been employed in developing a clinically useful assay in commercial production [29,30,31].

### 2.2. Liquid Biopsy Processing

PB samples (n = 3) were treated with isotonic ammonium chloride solution to lyse and remove the non-nucleated erythrocytes. The remaining cells were centrifuged, plated on custom 1-well adhesion slides (Marienfeld, Lauda, Germany), and stored at −80 °C as previously described [18,24,27,28,32].

CSF liquid biopsy samples (n = 3) were processed as follows. To estimate cellularity, 20 µL of sample was incubated on a single well of a 12-well adhesion slide (Marienfeld; Cat# 0900000) for 20 min at 37 °C. The suspension was removed, and PBS buffer added for brightfield imaging and manual enumeration of the cells present in the 20 µL CSF test sample to calculate the total number of cells in the volume of CSF provided. Prior to cell plating, the sample was centrifuged at 800 g for 5 min to collect the supernatant for cfDNA analysis. The cellular pellet was resuspended in PBS and plated onto 12-well or 3-well adhesion slide(s) (Marienfeld; Cat# 0900000 and 0901000), depending on the cellularity.

### 2.3. Immunofluorescent Staining and Automated Scanning

Blood slides were stained on an IntelliPATH FLX autostainer (Biocare Medical LLC, Pacheco, CA, USA) with positive and negative control slides according to the Landscape immunofluorescence (IF) staining protocol, as previously described [20,27,33]. CSF samples were stained manually using the same batch of reagents and protocol as the PB samples in the autostainer. Briefly, slides were fixed with 2% formalin, incubated with 10% goat serum, and stained with anti-mouse Fab fragments (100 μg/mL; IgG goat monoclonal; Jackson ImmunoResearch, West Grove, PA, USA; Cat# 115–007–003) and an anti-human CD31:Alexa Fluor 647 Antibody (2.5 μg/mL; mouse IgG1 monoclonal; Clone: WM59; BioRad, Hercules, CA, USA; Cat# MCA1738A647) prior to methanol permeabilization. Next, a cocktail of an anti-human vimentin (VIM): Alexa Fluor 488 antibody (3.5 μg/mL; rabbit IgG monoclonal; Clone: D21H; Cell Signaling Technology, Danvers, MA, USA; Cat# 9854BC), an anti-human CD45:Alexa Fluor 647 antibody (1.2 μg/mL; mouse IgG2a monoclonal; Clone: F10–89–4; AbD Serotec, Hercules, CA, USA; Cat# MCA87A647), an anti-human cytokeratin (CK) 19 antibody (0.2 μg/mL; mouse IgG1 monoclonal; Clone: RCK108; Dako, Santa Clara, CA, USA; Cat# GA61561–2), and an anti-human pan CK antibody mixture (210 μg/mL; CKs 1,4,5,6,8,10,13,18,19 mouse IgG1/IgG2a monoclonal; Clone: C-11, PCK-26, CY-90, KS-1A3, M20, A53-B/A2; Sigma, St. Louis, MO, USA; Cat# C2562) was incubated, followed by a mixture of 40,6-diamidino-2-phenylindole (DAPI; Dilution: 1: 50,000; Thermo Fisher Scientific, Waltham, MA, USA; Cat# D1306) and an anti-mouse IgG1: Alexa Fluor 555 secondary antibody (2 μg/mL; goat IgG polyclonal; Invitrogen, Carlsbad, CA, USA; Cat# A21127). A glycerol-based mounting medium was applied before the sample was coverslipped. The samples were imaged via automated fluorescence scanning microscopy (100× magnification) for DAPI (DNA, D), Alexa Fluor 488 (VIM), Alexa Fluor 555 (CK), and Alexa Fluor 647 (CD45/CD31, CD). Higher resolution images (400× magnification) were acquired manually for a subset of events.

### 2.4. Liquid Biopsy Analyte Detection and Classification

OCULAR, a rare event detection algorithm that includes statistical morphometric analysis of the single events from image extraction [20,25,27,33], was used for the PB analysis. Due to the low cellularity of normal CSF, and the importance in characterizing all cells present, every event was analyzed. Event classifications are based on the expression profile of the biomarkers (CK, VIM, CD) in the 4 fluorescent channels (DAPI, Alexa Fluor 488, Alexa Fluor 555, Alexa Fluor 647) and was done by trained analysts [25,27,33,34]. There were 8 cellular classifications defined by a nuclear structure which include CK-positive only CTCs (epi.CTCs), CK|VIM-positive CTCs (mes.CTCs), D|CK|CD, D|CK|VIM|CD, D-only, D|CD, D|VIM, and D|VIM|CD. Oncosomes (previously referred to as large extracellular vesicles) were defined as CK-positive circular events lacking a nuclear structure with variable VIM and CD expression [25,27,33,34].

### 2.5. Single Cell Genomic Analysis

Rare cell relocation, imaging, isolation, whole genome amplification (WGA), and copy-number alteration (CNA) analysis were conducted as previously described [17,18,20,28,32,35]. Cells of interest were relocated prior to individual cells being extracted using a robotic micromanipulator system. Isolated cells undergo single-cell WGA (Sigma-Aldrich; Cat# WGA4), library construction using the DNA Ultra Library Prep Kit (New England Biolabs, Ipswich, MA, USA; Cat# E7370), and low-pass sequencing using Illumina HiSeq at Fulgent Genetics. CNA profiles were constructed by mapping the reads from each individual cell to the human genome (hg19). Cells included in the analysis had total reads above 500,000 per cell, total alignment rate above 50%, nonsignificant noise, and no apoptosis-induced alterations. Ward.D clustering was used for hierarchical clustering to group genes or samples with similar expression patterns by iteratively joining clusters to minimize the increase in total variance (or within-cluster variance). The genomic instability (GI) score was calculated using a hyperbolic tangent function for the ratio to the median (RM) as previously reported [32]. The higher the GI score, the further the RM is from 1, and the more aberrant the CNA profile.

### 2.6. Targeted Multiplexed Proteomics Using IMC

Metal-labeled antibodies were procured from Standard Biotools (South San Francisco, CA, USA), either from the standard CyTOF catalog or as custom conjugates, and Abcam (Cambridge, UK). Metal-labeled antibody cocktails were prepared in 1% BSA, 0.1% Tween-20 in PBS, and sample staining was conducted as previously reported [20,34,35,36,37]. Briefly, samples were first blocked with 1% BSA in PBS and 0.2 mg/mL mouse IgG Fc fragment (Thermo Scientific) for 30 min, followed by incubation with the antibody cocktail for 1.5 h at room temperature and washing with PBS. Samples were then stained with cell membrane counterstain and DNA intercalator (Standard Biotools) for 30 min followed by another washing with PBS and a final rinse with ddH20 for 5 s. Samples were dried overnight at room temperature prior to ablation. A region of interest (ROI) with candidate cells was ablated with a 1 µm diameter pulsed laser, followed by ionization and detection on the CyTOF Helios instrument. Ion mass data were collected and used for reconstruction of the ROI at a 1 µm^2^ spatial resolution. Cell and nuclear borders were determined according to the method described in our prior publications [35]. Cells were segmented using ilastik ([38] v1.3.3) and single-cell masks were created in CellProfiler ([39] v2.2.0). ROIs were visualized by histocat++ [40]. Background ion counts from the negative mask space were subtracted from ion counts within the masked areas.

To visualize the ion counts for each antibody, the data for each were scaled by subtracting the mean and subsequently dividing by the standard deviation. The pheatmap (v1.0.12) package in R was used to visualize the results, with white indicating 0 (i.e., mean ion count), blue indicating negative values (i.e., below the mean), and red indicating positive values (i.e., above the mean).

### 2.7. MACSPlex Exosome Assay and Flow Cytometry Analysis

The exosome screening approach utilized the MACSPlex human Exosome Kit (Miltenyi, Bergisch Gladbach, Germany). The complete list of the 37 extracellular vesicle (EV) surface markers that were analyzed is represented in Supplemental Table S1. In total, 10 µL of centrifuged CSF from each patient were incubated with the MACSPlex capture beads overnight and washed with the MACSPlex Buffer solution prior to labeling with APC conjugated antibodies and analysis via flow cytometer. The MACSPlex Buffer was used as a blank control. Median fluorescence intensity (MFI) for each EV surface marker was normalized by the mean MFI for the negative control to provide a semi-quantitative MFI value per target.

## 3. Results

### 3.1. Patient Information

All three patients had leptomeningeal disease (LMD) on imaging that was not definitively confirmed by clinical CSF cytology. Patient 1 also had a brain metastasis, and clinical cytology indicated the presence of lymphocytes and monocytes, as well as atypical cells suspicious of a carcinoma. Patient 2 was on a prior treatment that controlled LMD, and no malignant cells were detected on clinical cytology. Patient 3 had highly atypical large mononuclear cells present in the CSF, suspicious for involvement with carcinoma. For all patients, disease was confined to the central nervous system.

### 3.2. Correlation of PB and CSF Liquid Biopsies

**Figure 2:**
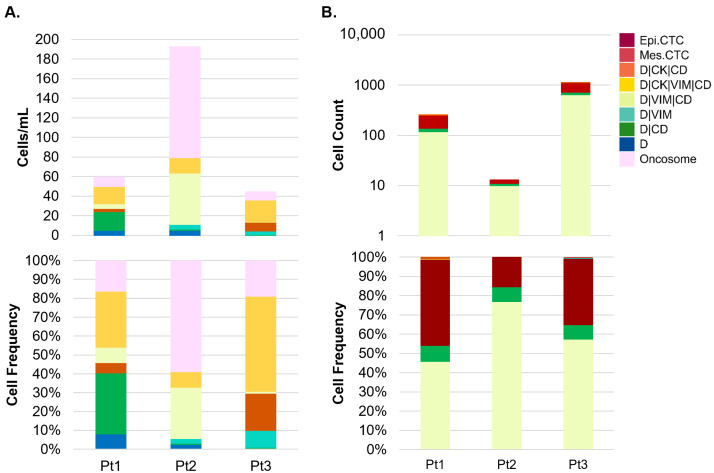
HDSCA3.0 of PB and CSF. (**A**) Counts (cell/mL) and frequency of rare cells found in the PB. (**B**) Counts (on log scale; total cells detected) and frequency of all cells identified in the CSF. Cellular classifications are based on the expression profile of biomarkers (CK, VIM, CD) in the four fluorescent channels (DAPI, Alexa Fluor 488, Alexa Fluor 555, Alexa Fluor 647), indicating positive expression relative to the background level. Epi.CTC = D|CK. Mes.CTC = D|CK|VIM.

**Figure 3:**
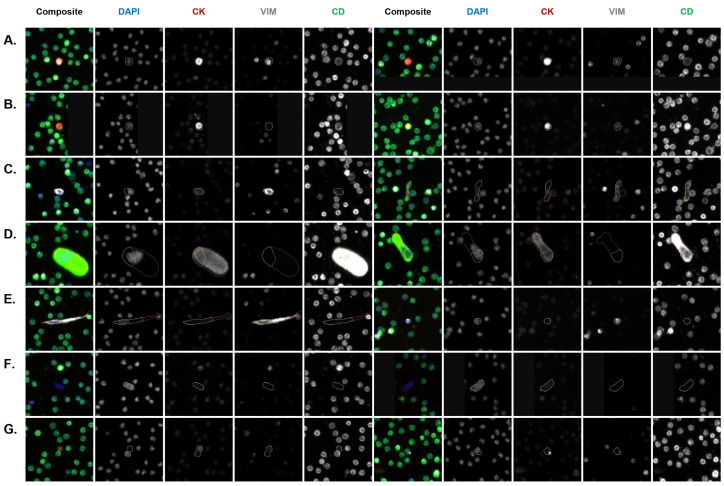
Representative gallery of rare events found in the PB across patients. (**A**) D|CK|VIM|CD, (**B**) D|CK|CD, (**C**) D|VIM|CD, (**D**) D|CD, (**E**) D|VIM, (**F**) D only, (**G**) oncosome. Cellular classifications are based on the expression profile of biomarkers (CK, VIM, CD) in the four fluorescent channels (DAPI, Alexa Fluor 488, Alexa Fluor 555, Alexa Fluor 647), indicating positive expression relative to the background level. Images taken at 100× magnification. The event of interest is located in the center of each image and marked by a cellular mask in each individual channel.

**Figure 4:**
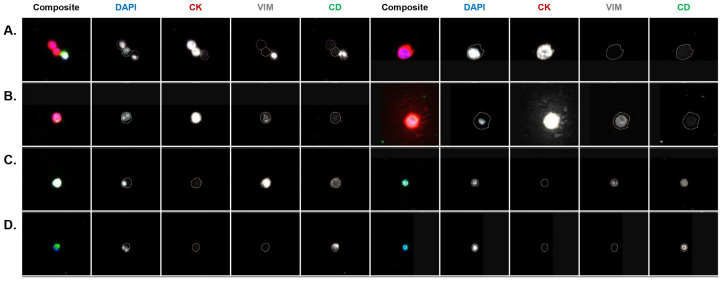
Representative gallery of cells detected in the CSF across patients. (**A**) epi.CTC, (**B**) mes.CTC, (**C**) D|VIM|CD, (**D**) D|CD. (**C**,**D**) are hypothesized WBCs ((**D**) left: multi-lobular nucleus presented in DAPI). Cellular classifications are based on the expression profile of biomarkers (CK, VIM, CD) in the four fluorescent channels (DAPI, Alexa Fluor 488, Alexa Fluor 555, Alexa Fluor 647), indicating positive expression relative to the background level. Epi.CTC = D|CK. Mes.CTC = D|CK|VIM. Images taken at 100× magnification. The event of interest is located in the center of each image and marked by a cellular mask in each individual channel. High-resolution images provided in Supplemental Figure S1.

Each PB sample was analyzed for the abundance and frequency of rare cells by fluorescent channel-type classification and oncosomes. The rare cell profile of the PB samples per patient is presented in Figure 2A. The total cellularity of the CSF samples is presented in Figure 2B. A total of 2, 3, and 4 mL of CSF was analyzed for Patient 1, 2, and 3, respectively. Figure 3 and Figure 4 show a representative gallery of cells in each compartment. In all three patients, epi.CTCs and mes.CTCs were detected in the CSF, but not the PB. The PB presented with a heterogeneous population of rare cells. Oncosomes were additionally detected in the PB in all three patients (9.62, 113.37, 8.36 oncosomes/mL for Patients 1, 2, and 3, respectively). Each patient presented with a unique rare event profile in the PB. In Patient 2’s PB, the oncosomes made up 59% of the rare event profile. The D|CD cells dominated in Patient 1 (32%), while Patient 3 presented with 51% D|CK|VIM|CD cells.

In the CSF, the cellular population consisted mainly of epi.CTCs (range 15–44%), D|CD (range 7.5–8.1%), and D|VIM|CD (range 46–77%) cells (Figure 2B). The CSF epi.CTCs were morphologically larger than the surrounding D|CD and D|VIM|CD cells in the sample, and presented as either individual cells or clusters (Supplemental Figure S1). The D|CD and D|VIM|CD cells are hypothesized to be white blood cells (WBCs) given their circular morphology, small size compared to the epi.CTCs and mes.CTCs, a majority exhibiting multi-lobular nuclei, and what appears to be a homogenous expression in the Alexa Fluor 647 channel characteristic of CD45. Patient 1 presented with the highest frequency of epi.CTCs, while Patient 2 had the lowest. Interestingly, the detection of epi.CTCs in the CSF was negatively correlated with the oncosomes detected in the PB (r2 = −0.99; *p*-value = 0.0119; Figure 2).

### 3.3. CSF Exosome Analysis

**Figure 5:**
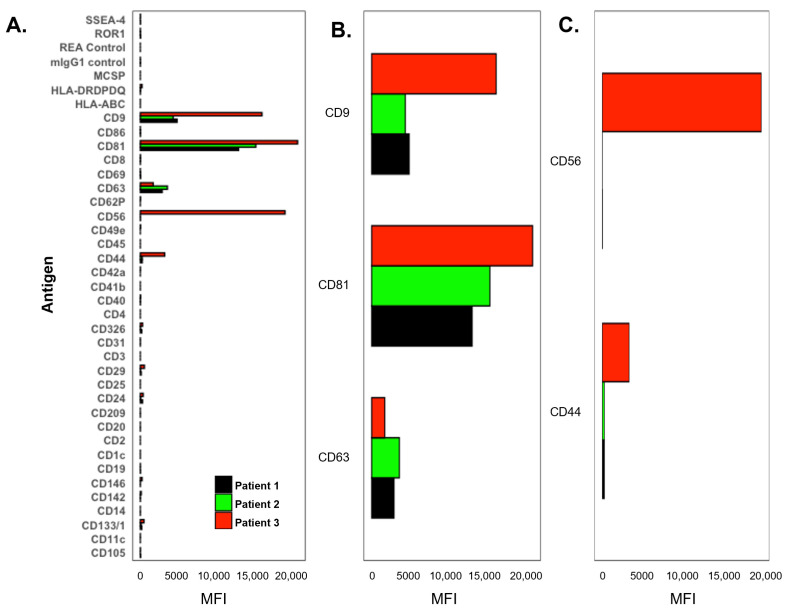
MACSPlex extracellular vesicle (EV) surface marker analysis. (**A**) Semi-quantitative signal of (**A**) total, (**B**) tetraspanin, and (**C**) adhesion biomarkers normalized to the negative control by patient. MFI = mean fluorescence intensity. Patients are color-coded.

The MACSPlex EV surface marker analysis enables the characterization of 37 distinct EV subpopulations in CSF (Figure 5A). Analysis of CSF samples from the three presented patients revealed a prominent signal for all three tetraspanins (CD9/CD63/CD81), with CD81 exhibiting the highest signal across all patients (Figure 5B). This observation aligns with previous findings reporting that CD81 and CD9 are more abundant than CD63 in CSF.

In Patient 3, a higher overall number of signals were detected (66,988 total EV count) compared to the other two patients (Patient 1: 23,242, Patient 2: 25,159) (Supplemental Table S1). Notably, CD56 (20,219 for Patient 3 vs. 18 and 0 for Patient 1 and 2, respectively) and CD44 (3402 vs. 265 and 257) exhibited significantly elevated levels beyond a proportional scale, suggesting that they could serve as signature markers specific to the patient case or disease status (Figure 5C).

### 3.4. Molecular Characterization of CSF CTCs

Single-cell and bulk cfDNA genomics and targeted multiplexed proteomics were conducted on the CSF sample collected from Patient 1.

**Figure 6:**
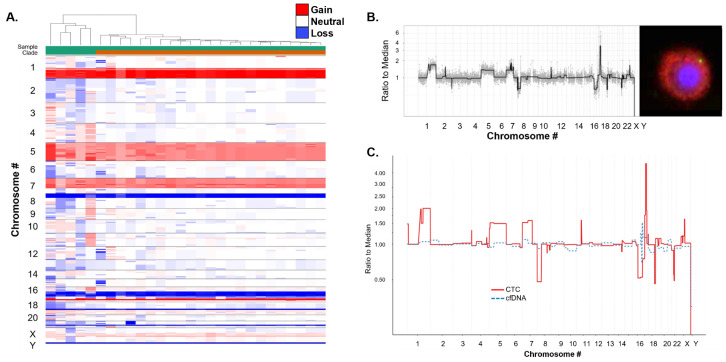
Genomic analysis of CSF. (**A**) Heatmap and phylogenic tree of single-cell CNAs across the entire population of cells isolated. Each column represents an individual CTC or cfDNA profile and each row shows chromosomal regions. For each sample, segments are shown as colored rectangles, where colors indicate copy number changes: white for neutral, red for gains, and blue for losses. The color intensity reflects the difference between the copy number and set thresholds (upper limit 1.25, lower limit 0.75). (**B**) Single epi.CTC CNA profile with corresponding fluorescent image at 400× magnification. (**C**) Comparison of CNAs from cfDNA and a single epi.CTC.

Single-cell genomic analysis was conducted on 28 CK+ (epi.CTCs) from the CSF sample. All epi.CTCs (100%) were identified to have genomic alterations that comprise a clonal population of cells (Figure 6). This clonal architecture was not identified in the cfDNA, and no tumor fraction was detectable (Figure 6C). The GI score of each cell is provided in Supplemental Table S2. For all epi.CTCs, there was a median of 590.05 (range 320.6–999.6, mean 603.41). The major aberrations included gains in 1q (GI chr 1 median 159.37), 5 (GI chr 5 median 90.84), 7p (GI chr 7 median 57.53), and 17q (GI chr 17 median 81.24), and losses in 8p (GI chr 8 median 76.59), 16q (GI chr 16 median 49.08), 17p (GI chr 17 median 81.24), and 18q (GI chr 18 median 23.04). The clonal population had a focal gain in chromosome 17 corresponding with ERBB2, which is likely the cause of HER2 overexpression.

**Figure 7:**
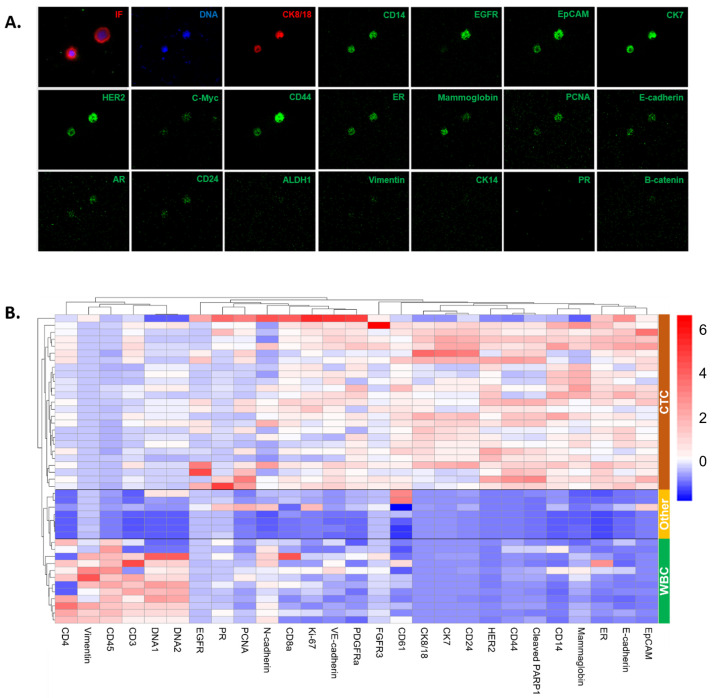
Multiplexed targeted proteomic analysis. (**A**) IF and representative image mass cytometry images of CTCs detected in the CSF. (**B**) Heatmap of targeted protein expression on cells detected in the CSF with hierarchical clustering. The heat map uses blue for low values, white for neutral, and red for high values, showing data intensity from cool (low) to warm (high). A total of 44 cells: 24 CTCs, 20 WBCs and other cells.

For this study, a panel of metal-labeled antibodies was designed to include targets relevant to BC, as well as immune cell markers. Targeted multiplexed proteomic analysis was conducted on seven ROIs using a biomarker panel of 36 antibodies and two DNA intercalators. A total of 44 cells were analyzed, with 24 of those cells classified as CTCs. The high degree of clonality in the CNV profiled cells provided assurance that the candidate CTCs selected for subsequent IMC analysis were likely to be bona fide cancer cells from the same lineage. However, we note that despite sharing the same genotype, individual cells exhibit significant phenotypic variability. The results of targeted proteomic analysis by imaging mass cytometry (IMC) are presented as representative CTC images from an example ROI (Figure 7A) and an expression heatmap (Figure 7B). Eleven protein targets were removed from the visualization due to neutral expression across all cells. A heatmap with the full panel of targets is presented in Supplementary Figure S2.

The proteomic results on Epi.CTCs in the CSF show that CK7 and CK8/18 expression was positive, while VIM expression was negative, confirming and complementing the IF results. It is important to note that the IF pan-CK cocktail included more CK proteins (1, 4, 5, 6, 8, 10, 13, 18, 19) than those targeted in the IMC panel, though proteomic analysis did suggest the CTCs to be negative in CK14, which is not part of the IF cocktail of antibodies. The CTCs have variable expression for the epithelial and cancer-specific markers, such as EpCAM, EGFR, E-cadherin, mammaglobin, HER2, ER, and AR. Further, the CTCs were negative for the immune markers (CD45, CD4, CD3, CD14). A subpopulation of the CTCs is CD44 high and CD24 low, which is associated with stem cell-like and invasive features.

The non-CTCs detected in the CSF were phenotypically stratified into two main categories: (1) WBCs, which were positive for the immune markers and negative for the epithelial and cancer-specific markers, and (2) other cells with minimal to no expression of the target proteins. The WBCs were specifically positive for CD45, CD3, and CD4, which suggest they are T regulatory cells. Interestingly, there were distinct DNA signal intensities observed by cell type. This is likely due to variable nuclear sizes in which the DNA signal is denser in the WBCs than in the CTCs.

## 4. Discussion

The liquid biopsies (PB and CSF) from three patients with breast to brain metastases were analyzed using the HDSCA3.0 workflow. Importantly, CTCs were detected in the CSF samples using the HDSCA3.0 workflow but not by standard clinical CSF cytological analysis. Further, CTCs were not detected in the PB, and molecular characterization by integrating proteomics via IMC and genomic data (CNV) allowed for the characterization of CSF CTCs. From the analysis of Patient 1’s CSF, we detected the presence of a genomically clonal population of CTCs with a heterogeneous phenotype suggesting cellular plasticity. The variable expression of protein markers such as EpCAM on the CSF CTCs suggests biomarker enrichment-based methodologies would miss a subset of cells potentially important in characterizing the disease to inform clinical decision making. The data presented suggest that the HDSCA3.0 workflow may be used to measure and characterize disease burden in the CSF to diagnose disease progression or inform treatment decision making.

We further analyzed the CSF to investigate the surface-antigen profile of EVs using a screening approach that explored 37 unique surface markers. EVs are nano-sized particles secreted by cells that play an important role in intercellular communication and numerous biological mechanisms, such as immune responses, inflammation, and coagulation processes [41,42]. Tumor-derived EVs have been associated with the development of distant metastases through the reprogramming of cells to support entry in the pre-metastatic niche [43,44]. The observation that CD81 exhibited the highest signal across all patients in this study aligns with previous findings reported in CSF [45,46]. Many studies have identified higher levels of specific EVs in the CSF that are associated with the diagnosis and prognosis of stroke [47,48,49]; however, the data are limited regarding patients with brain metastases. The MACSPlex analysis revealed patient-specific differences with signal from immune/inflammation-related and epithelial (CTCs) cells, as well as cancer-related markers (CD24, CD29, CD44, and CD146), which represent a valuable source of information to further understand the mechanisms of cancer biology in brain metastases.

Studies have demonstrated the potential of circulating tumor DNA (ctDNA) analysis in PB, but for brain tumors it is more challenging due to factors such as low levels of ctDNA in plasma, which may be blocked by the blood–brain barrier from exiting the central nervous system. Therefore, in brain tumor patients, the use of CSF ctDNA for liquid biopsy is a promising alternative to PB, with studies showing an increase in concentration and improved detection rates [50,51,52,53,54]. In the patient analyzed here, we observed a lack of CNA profile concordance between cfDNA and single cells detected in the CSF. Further analysis in additional patients is warranted to understand the correlation between cells and cfDNA in the CSF. It is important to note that analysis of cfDNA includes detection of ctDNA from various tumor cells and healthy cfDNA. By using the acellular fraction as a repository for genomic alterations, we may be able to monitor the overall tumor evolution in response to therapy. Beyond the cfDNA, unique to the study presented here is the corresponding genomic analysis of the single CTCs. Unlike a regular tissue biopsy of a single lesion, which may not produce a complete profile of mutations, the liquid biopsy can provide both cellular and acellular information for bulk and single-cell comparison.

Despite the lack of CTCs present in the PB, oncosomes were identified in the systemic circulation. In a previous publication, we reported the presence of oncosomes as a significant circulating analyte in distinguishing early-stage from late-stage BC and normal donors [27], indicating that oncosomes are present prior to CTCs in the evolution of disease. Tumor-derived oncosomes, released from either the primary or metastatic lesion, may impact the initiation and propagation of cancer. In this study, the disease is confined to the central nervous system, which may explain the lack of CTCs in systemic circulation. Studies have shown that EVs can cross the blood–brain barrier [55,56], suggesting that the presence of oncosomes in the PB may be due to passage across or disruption of the blood–brain barrier. Further research is warranted to understand the significance of oncosomes in the liquid biopsy and their role in metastasis.

The main limitation of this pilot study is the number of patients included, and thus a larger cohort is needed for validation. Due to the nature of the HDSCA3.0 workflow, we have the unique advantage of analyzing the full complement of cells in the liquid biopsy, enabling immunophenotyping alongside CTC characterization. Utilizing multiplexed proteomics with a targeted panel allows for the interrogation of cancer-specific markers and immune markers, as well as markers for discovery and exploration across cell states and biological mechanisms. Patient-specific immune signatures, in addition to the cancer cells, could potentially provide insight into the classification and monitoring of cancers for precision medicine. Future work will examine the utility of this CSF approach in treatment monitoring, correlating findings with standard clinical procedures, while also analyzing CSF analytes from different brain and spinal regions of patients with LMD or brain metastases. We aim to validate our pilot study findings in larger clinical studies, necessitating specifically designed studies to assess them at scale. Key challenges include the limited patient population for rare diseases and ensuring consistent sample collection across sites, which can be addressed through standardized protocols and broad institutional collaboration to pool data.

## 5. Conclusions

Conventional CSF cytology is neither quantitative nor does it allow for the characterization of the tumor cells, limiting clinicians to a binary judgment call based on the presence or absence of malignant cells. In this pilot study of three patients, we show a qualitative and quantitative assessment with the potential of molecular characterization to open new insights for patients with brain metastases by tailoring therapeutic strategies and monitoring treatment efficacy. The CSF liquid biopsy analysis by HDSCA3.0 has the potential to advance clinical care by enabling (1) molecular diagnosis when tumor tissue is not readily accessible, (2) longitudinal monitoring of disease response to treatment, and (3) tracking tumor evolution throughout the course of disease to uncover new insights into tumor biology, as well as (4) informing clinical trial enrollment and (5) providing prognostication at various disease stages. Guided CSF monitoring could significantly enhance patient care and outcomes.

## Data Availability

All data discussed in this manuscript are included in the main manuscript text or Supplementary Materials. The imaging data are available through the BloodPAC Data Commons, Accession ID “BPDC000143” (https://data.bloodpac.org/discovery/BPDC000143/ (accessed on 30 October 2024)).

## Supplementary Materials

The following supporting information can be downloaded at: https://www.mdpi.com/article/10.3390/cancers16223746/s1, Table S1: MACSPlex EV analysis of the CSF patient samples. Semi-quantitative signals of each biomarker normalized to the negative control; Figure S1: Representative high-resolution images of cells detected in the CSF. Images taken at 400× magnification; Figure S2: Multiplexed targeted proteomic analysis. Heatmap of targeted protein expression on cells detected in the CSF with hierarchical clustering. Proteomic panel consisted of 36 antibodies and 2 DNA intercalators. Total 44 cells: 24 CTCs, 20 WBCs and Other cells; Table S2: Genomic instability score of single cells sequenced with descriptive statistics. The GI score is provided for the whole genome and each individual chromosome; Table S3. Glossary of terminology.
