# Supplementary material for: Multi-Omic Characterization of Single Cells and Cell-Free Components Detected in the Cerebrospinal Fluid of Patients with Leptomeningeal Disease
Source: Cancers (Basel). 2024 Nov 6;16(22):3746. doi: 10.3390/cancers16223746 (PMC11592257; doi:10.3390/cancers16223746)
Supplement: Supplementary file 1 [file cancers-16-03746-s001.zip › cancers-3231460-supplementary.pdf]

Table S1. MACSPlex EV analysis of the CSF patient samples. Semi-quantitative signals of each biomarker normalized to the negative control.

Antigen	Patient 1	Patient 2	Patient 3
CD3	0	0	0
CD4	0	0	0
CD19	21	0	9
CD8	6	22	20
HLA-DRDPDQ	9	28	241
CD56	18	0	20219
CD105	47	0	16
CD2	0	0	0
CD1c	0	0	0
CD25	0	0	0
CD49e	0	0	37
ROR1	40	19	8
CD209	4	0	0
CD9	5123	4589	16998
SSEA-4	25	0	20
HLA-ABC	0	0	0
CD63	3050	3781	1788
CD40	15	9	42
CD62P	0	0	28
CD11c	0	0	5
CD81	13718	16140	21975
MCSP	0	0	0
CD146	0	7	246
CD41b	0	0	0
CD42a	0	0	14
CD24	302	62	398
CD86	19	21	2
CD44	265	257	3402
CD326	201	22	309
CD133/1	195	123	511
CD29	124	33	570
CD69	39	23	4
CD142	7	1	126
CD45	0	0	0
CD31	0	7	0
REA Control	0	0	0
CD20	2	0	0
CD14	4	0	0
mIGG1 control	8	15	0
Total	**23242**	**25159**	**66988**

Table S2. Genomic instability score of single cells sequenced with descriptive statistics. The GI score is provided for the whole genome and each individual chromosome.

Cell#	GI Score	Variance	Chr1	Chr2	Chr3	Chr4	Chr5	Chr6	Chr7	Chr8	Chr9	Chr10	Chr11	Chr12	Chr13	Chr14	Chr15	Chr16	Chr17	Chr18	Chr19	Chr20	Chr21	Chr22	X/Y
1	595.30	1.70	142.20	30.24	8.44	13.28	45.32	3.35	69.90	94.21	3.07	7.24	11.14	4.78	8.90	3.26	0.81	37.58	73.70	12.47	4.70	1.06	3.88	8.73	7.04
2	609.10	2.30	169.47	0.78	1.26	3.14	94.91	1.12	63.56	89.06	0.30	0.50	2.20	0.40	1.23	0.72	0.41	48.95	85.50	23.40	2.54	0.14	0.25	14.64	4.64
3	610.10	2.50	163.17	3.45	0.32	3.03	79.88	1.35	54.74	70.02	1.63	2.97	3.83	0.29	1.45	1.04	0.08	50.64	65.26	20.23	4.11	1.58	64.12	12.01	4.93
4	733.50	1.90	172.69	3.63	4.76	2.09	86.64	23.54	67.37	71.29	4.87	1.64	4.22	32.64	2.05	3.46	55.23	48.76	77.09	25.70	1.80	8.80	11.86	17.52	5.82
5	672.80	2.00	138.42	12.37	7.64	14.20	99.54	8.08	51.72	104.28	3.18	5.48	6.07	9.52	4.58	5.92	0.00	73.85	71.21	26.48	5.45	1.52	2.97	13.35	7.01
6	592.90	1.90	159.87	1.12	0.10	1.32	94.59	1.97	59.36	75.65	0.25	0.97	2.18	0.28	0.12	1.68	0.21	55.68	91.16	24.71	1.02	0.01	0.38	13.44	6.80
7	586.30	1.90	140.61	6.30	2.58	3.96	106.78	7.87	48.43	69.51	1.91	0.48	3.02	2.68	4.65	0.92	2.33	45.99	84.28	25.05	1.41	3.61	0.22	17.33	6.38
8	654.50	2.00	149.19	10.95	2.26	34.17	118.01	4.96	59.68	42.92	24.75	1.37	44.52	0.67	2.50	0.45	0.00	23.20	85.25	26.67	2.16	0.80	0.65	14.37	5.02
9	581.30	1.90	159.08	2.70	2.17	1.69	89.82	2.43	54.12	79.92	0.58	1.06	1.79	0.60	0.89	1.07	0.63	52.36	80.68	22.66	3.36	1.11	0.03	16.60	5.91
10	598.50	2.50	159.66	3.32	3.77	0.92	96.95	2.68	58.15	89.96	0.64	2.84	5.23	2.81	0.94	2.75	1.38	41.51	77.24	19.86	1.74	0.00	0.32	10.71	5.73
11	999.60	2.10	155.92	25.27	33.31	11.22	228.78	29.78	86.88	134.03	9.43	7.58	11.19	10.91	5.70	13.37	7.82	17.76	117.40	45.42	10.85	14.63	3.59	12.95	5.83
12	596.60	1.80	166.14	0.76	4.00	1.33	85.42	1.83	76.23	87.11	1.99	0.64	1.83	0.43	2.29	1.30	0.98	42.74	75.26	24.86	2.98	0.33	0.29	12.23	5.65
13	476.90	1.80	135.10	0.27	1.93	1.46	79.26	0.72	55.63	56.72	0.80	0.56	2.06	1.30	1.69	0.16	0.19	37.63	75.22	10.90	1.45	1.11	0.19	8.50	4.09
14	586.80	1.80	165.29	1.60	0.25	0.27	90.46	1.46	59.11	83.60	0.27	0.72	2.26	0.59	1.09	0.25	0.01	47.71	84.36	25.31	1.94	0.27	0.13	14.86	4.98
15	587.20	1.80	160.74	3.18	0.73	0.13	94.52	1.15	57.84	74.02	0.36	1.63	1.86	0.29	1.30	0.62	1.16	59.17	81.80	22.69	1.39	0.52	0.29	16.09	5.75
16	578.60	2.20	161.27	1.19	1.33	3.89	91.16	1.64	59.08	74.04	0.24	0.60	0.46	1.27	0.52	0.09	0.54	51.17	83.41	22.17	2.56	0.21	0.04	17.01	4.72
17	649.70	2.10	198.56	6.81	3.92	1.36	88.31	3.41	59.65	67.00	5.15	5.74	2.64	13.01	1.91	1.68	1.57	57.53	87.83	20.37	2.89	0.52	0.14	14.95	4.76
18	606.20	2.10	165.47	3.21	1.45	1.87	98.18	0.79	54.73	85.16	1.07	2.52	2.05	1.37	1.01	1.17	0.84	54.12	82.47	25.25	2.33	0.93	0.10	14.77	5.31
19	569.60	2.00	152.81	3.34	0.16	1.42	85.23	1.14	52.15	75.08	0.11	1.00	2.09	1.15	2.19	0.05	0.24	55.71	87.11	20.94	2.40	0.42	0.01	19.88	4.99
20	583.60	2.00	162.82	4.64	0.65	0.95	87.28	1.46	56.32	76.61	1.14	3.58	1.66	1.40	1.52	1.78	0.10	49.22	84.38	25.84	2.51	0.45	0.72	13.00	5.49
21	559.90	2.00	148.25	3.14	0.22	0.54	87.09	2.15	62.88	76.17	1.13	1.03	4.40	1.47	1.42	1.58	0.77	42.32	80.69	22.43	2.41	0.31	0.44	13.02	6.06
22	586.60	2.10	167.64	1.05	2.20	0.25	92.41	1.51	56.37	71.59	0.49	3.00	3.06	1.10	1.51	0.89	0.08	49.64	82.89	25.61	1.38	1.59	0.87	14.92	6.54
23	553.30	2.00	151.32	2.62	2.18	0.56	90.52	1.39	53.76	80.75	0.79	1.42	2.87	0.92	0.00	0.35	0.03	43.84	75.19	25.67	2.40	0.40	0.01	9.70	6.62
24	568.50	2.10	154.30	0.29	0.35	2.24	99.73	1.94	56.90	76.57	0.20	1.31	3.31	0.95	1.20	1.36	0.20	54.60	72.47	20.92	2.46	0.10	0.09	11.75	5.21
25	628.00	2.10	164.06	6.20	2.59	5.13	103.72	2.83	57.22	97.54	2.00	2.30	2.53	3.38	2.62	2.32	0.00	48.90	76.86	22.02	3.19	0.56	1.28	13.91	6.81
26	320.60	1.80	86.93	4.63	0.85	1.97	54.95	1.92	38.30	29.56	1.91	0.75	2.23	2.92	0.00	0.44	0.94	22.98	47.90	8.15	3.39	0.19	0.14	4.88	4.67
27	565.00	1.90	143.26	3.38	0.35	2.59	93.39	0.94	58.27	82.00	0.22	0.93	1.71	0.48	0.16	0.14	0.10	52.23	81.87	22.62	1.82	0.14	0.01	14.05	4.34
28	644.60	2.20	130.80	12.91	7.21	8.23	80.54	6.57	30.82	135.01	5.86	10.32	8.85	6.08	6.58	4.31	2.67	66.34	54.02	38.49	4.97	3.79	2.10	13.80	4.32
**average**	603.41	2.02	154.47	5.69	3.46	4.40	94.41	4.29	57.83	80.33	2.66	2.51	5.05	3.70	2.14	1.90	2.83	47.58	79.38	23.46	2.91	1.61	3.40	13.53	5.55
**min**	320.60	1.70	86.93	0.27	0.10	0.13	45.32	0.72	30.82	29.56	0.11	0.48	0.46	0.28	0.00	0.05	0.00	17.76	47.90	8.15	1.02	0.00	0.01	4.88	4.09
**max**	999.60	2.50	198.56	30.24	33.31	34.17	228.78	29.78	86.88	135.01	24.75	10.32	44.52	32.64	8.90	13.37	55.23	73.85	117.40	45.42	10.85	14.63	64.12	19.88	7.04
**median**	590.05	2.00	159.37	3.33	2.05	1.92	90.84	1.93	57.53	76.59	1.10	1.39	2.58	1.29	1.48	1.12	0.47	49.08	81.24	23.04	2.43	0.52	0.29	13.85	5.57

Table S3. Glossary of terminology.

Term	Definition
Blood-Brain Barrier	A selective barrier that prevents many substances from entering the brain, often posing a challenge for liquid biopsies of CNS tumors due to limited ctDNA and CTC access.
Biophysical Enrichment	Techniques such as size filtration or magnetic bead separation used to isolate specific cells based on physical characteristics.
Cell-Free DNA (cfDNA)	DNA fragments released into body fluids from cells, including cancer cells; used as a biomarker in liquid biopsies to detect tumor-related genetic alterations.
Cerebrospinal Fluid (CSF)	A clear, colorless body fluid found in the brain and spinal cord, used in this study as a liquid biopsy medium to monitor central nervous system (CNS) disease progression.
Circulating Tumor Cells (CTCs)	Cancer cells that have detached from the primary or metastatic tumor and circulate in the bloodstream or CSF, used for diagnostic and prognostic analysis.
Clonality	The origin of a population of cells from a single progenitor, relevant in cancer to determine if tumor cells share genetic alterations, suggesting they arise from a common source.
Copy Number Alteration (CNA)	Variations in the DNA structure that lead to the gain or loss of copies of DNA sections from a normal genome. The number of copies of a particular gene or genomic region are presented, indicative of genetic instability in cancer cells.
Epithelial cell adhesion molecule (EpCAM)	A transmembrane glycoprotein mediating Ca -independent homotypic cell–cell adhesion in epithelia and involved in cell signaling, migration, proliferation, and differentiation. Often overexpressed on the surface of epithelial cancer cells, used as a biomarker for detecting CTCs.
Extracellular Vesicles (EVs)	Nano-sized particles released by cells, including cancer cells, involved in intercellular communication and potential indicators of disease.
Fluorescence Imaging	A technique used to visualize specific cell components labeled with fluorescent markers, essential for identifying rare events such as CTCs in the HDSCA3.0 workflow.
Genomic Instability (GI) Score	A measure of the extent of CNA within a single cell, indicating the cell's genetic instability—a hallmark of cancer cells.
HER2-Positive	HER2, short for human epidermal growth factor receptor 2, describes a protein that plays a role in normal breast cell development. This refers to cancer cells with an overexpression of the HER2 protein, commonly associated with more aggressive breast cancer.
High-Definition Single Cell Assay (HDSCA3.0)	A non-enrichment workflow used to analyze single cells and cell-free components in liquid biopsies without the need for initial enrichment steps.
Imaging Mass Cytometry (IMC)	A technology that combines imaging and mass spectrometry to detect multiple protein markers in a single tissue or cell sample.
Leptomeningeal Disease (LMD)	A condition where cancer spreads to the leptomeninges, the membranes surrounding the brain and spinal cord, often complicating prognosis and treatment.
Liquid Biopsy	A minimally invasive method to detect cancer-related biomarkers (cells, DNA, or extracellular vesicles) in body fluids, providing an alternative to tissue biopsies.
Mesenchymal CTCs (mes.CTCs)	A subtype of CTCs expressing mesenchymal markers, often associated with metastatic potential and increased aggressiveness.
Multi-Omic Analysis	The simultaneous study of multiple "omics" layers (e.g., genomics, proteomics) to gain a comprehensive understanding of biological phenomena.
Peripheral Blood (PB)	Flowing, circulating blood of the body, which includes erythrocytes (red blood cells), leukocytes (white blood cells), and thrombocytes (platelets) suspended in blood plasma. It circulates through the veins and arteries in the arms, hands, legs, and feet. Here PB is used as a liquid biopsy to analyze cancer-related biomarkers outside the CNS.
OCULAR	A rare event detection algorithm used in the study for statistical morphometric analysis, helping identify and classify rare cells in blood samples.
Oncosomes	Large extracellular vesicles derived from cancer cells, often carrying tumor-specific proteins and genetic material, used as biomarkers for early-stage or metastatic cancer.
Rickham Reservoir	A surgically implanted device connected to the lateral ventricle of the brain, allowing for repeated CSF sampling and intrathecal administration of drugs.
Spatial Resolution	The level of detail that can be distinguished in an image, significant in high-resolution fluorescence and mass cytometry imaging used to characterize single cells.
Tetraspanins	A family of four-pass transmembrane proteins (e.g., CD9, CD63, CD81) that regulate cellular functions by interacting with diverse partner proteins and are detectable on the surface of EVs. Here they are used to characterize EV subpopulations in liquid biopsy samples.
Tumor Microenvironment	The environment around a tumor, including surrounding blood vessels, immune cells, and extracellular molecules, influencing cancer progression and response to treatment.
Whole Genome Amplification (WGA)	A technique used to amplify the entire genome from a single cell, facilitating downstream genomic analyses like CNA profiling.

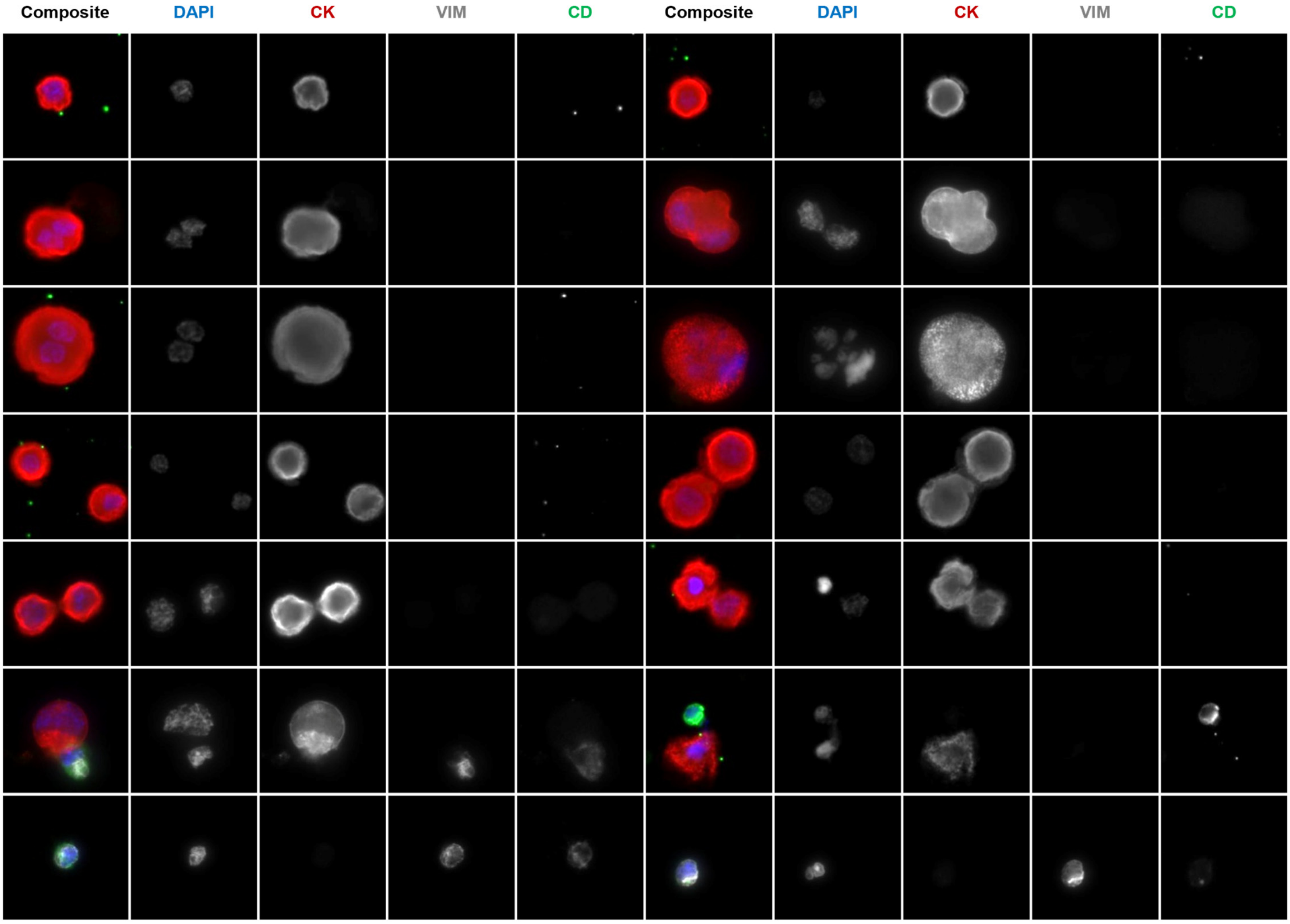

Figure S1. Representative high-resolution images of cells detected in the CSF. Images taken at 400x magnification.

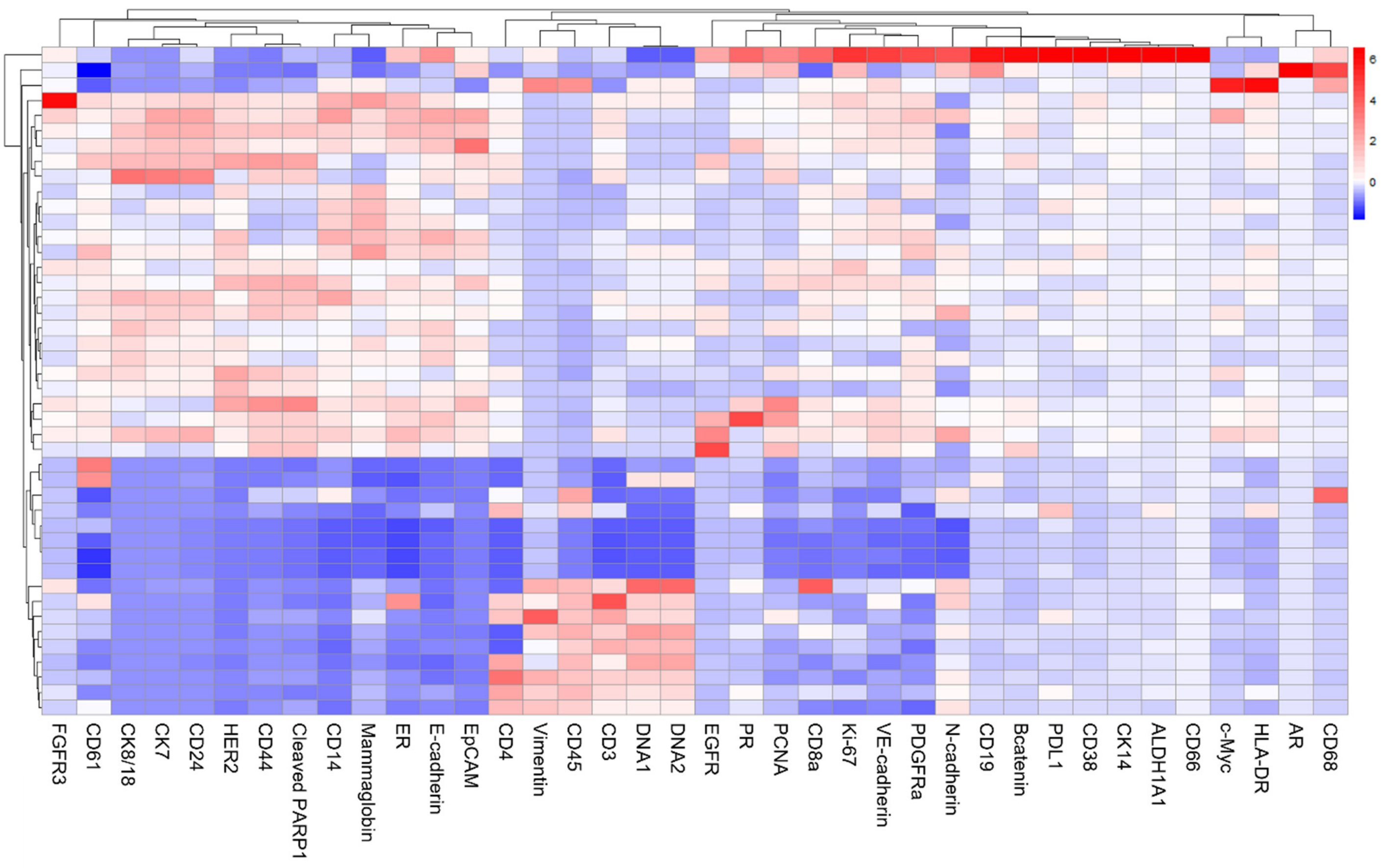

Figure S2. Multiplexed targeted proteomic analysis. Heatmap of targeted protein expression on cells detected in the CSF with hierarchical clustering. Proteomic panel consisted of 36 antibodies and 2 DNA intercalators. Total 44 cells: 24 CTCs, 20 WBCs and Other cells.
